# Supplementary material for: Resolving the full spectrum of human genome variation using Linked-Reads
Source: Genome Res. 2019 Apr;29(4):635–45. doi: 10.1101/gr.234443.118 (PMC6442396; doi:10.1101/gr.234443.118)
Supplement: Supplemental Material [file supp_gr.234443.118_Supplemental_longranger-2.2.zip › longranger-2.2.tar.bz2!/lr2.2-public/lib/stan/cmdstan/stan/doc/api/html/get__base1_8hpp_source.html]

Stan: src/stan/math/matrix/get\_base1.hpp Source File


|  |  |
| --- | --- |
|  | Stan  2.5.0  probability, sampling & optimization |


- Main Page
- Related Pages
- Namespaces
- Classes
- Files

- File List
- File Members

All Classes Namespaces Files Functions Variables Typedefs Enumerations Enumerator Friends Macros Pages

- src
- stan
- math
- matrix

get\_base1.hpp

Go to the documentation of this file.

1 #ifndef STAN\_\_MATH\_\_MATRIX\_\_GET\_BASE1\_HPP

2 #define STAN\_\_MATH\_\_MATRIX\_\_GET\_BASE1\_HPP

3

4 #include <vector>

5 #include <stan/math/matrix/Eigen.hpp>

6 #include <stan/math/error\_handling/matrix/check\_range.hpp>

7

8 namespace stan {

9  namespace math {

10

25  template <typename T>

26  inline const T&

27  get\_base1(const std::vector<T>& x,

28  size\_t i,

29  const char\* error\_msg,

30  size\_t idx) {

31  check\_range(x.size(),i,error\_msg,idx);

32  return x[i - 1];

33  }

34

50  template <typename T>

51  inline const T&

52  get\_base1(const std::vector<std::vector<T> >& x,

53  size\_t i1,

54  size\_t i2,

55  const char\* error\_msg,

56  size\_t idx) {

57  check\_range(x.size(),i1,error\_msg,idx);

58  return get\_base1(x[i1 - 1],i2,error\_msg,idx+1);

59  }

60

77  template <typename T>

78  inline const T&

79  get\_base1(const std::vector<std::vector<std::vector<T> > >& x,

80  size\_t i1,

81  size\_t i2,

82  size\_t i3,

83  const char\* error\_msg,

84  size\_t idx) {

85  check\_range(x.size(),i1,error\_msg,idx);

86  return get\_base1(x[i1 - 1],i2,i3,error\_msg,idx+1);

87  }

88

106  template <typename T>

107  inline const T&

108  get\_base1(const std::vector<std::vector<std::vector<std::vector<T> > > >& x,

109  size\_t i1,

110  size\_t i2,

111  size\_t i3,

112  size\_t i4,

113  const char\* error\_msg,

114  size\_t idx) {

115  check\_range(x.size(),i1,error\_msg,idx);

116  return get\_base1(x[i1 - 1],i2,i3,i4,error\_msg,idx+1);

117  }

118

137  template <typename T>

138  inline const T&

139  get\_base1(const std::vector<std::vector<std::vector<std::vector<std::vector<T> > > > >& x,

140  size\_t i1,

141  size\_t i2,

142  size\_t i3,

143  size\_t i4,

144  size\_t i5,

145  const char\* error\_msg,

146  size\_t idx) {

147  check\_range(x.size(),i1,error\_msg,idx);

148  return get\_base1(x[i1 - 1],i2,i3,i4,i5,error\_msg,idx+1);

149  }

150

170  template <typename T>

171  inline const T&

172  get\_base1(const std::vector<std::vector<std::vector<std::vector<std::vector<std::vector<T> > > > > >& x,

173  size\_t i1,

174  size\_t i2,

175  size\_t i3,

176  size\_t i4,

177  size\_t i5,

178  size\_t i6,

179  const char\* error\_msg,

180  size\_t idx) {

181  check\_range(x.size(),i1,error\_msg,idx);

182  return get\_base1(x[i1 - 1],i2,i3,i4,i5,i6,error\_msg,idx+1);

183  }

184

185

206  template <typename T>

207  inline const T&

208  get\_base1(const std::vector<std::vector<std::vector<std::vector<std::vector<std::vector<std::vector<T> > > > > > >& x,

209  size\_t i1,

210  size\_t i2,

211  size\_t i3,

212  size\_t i4,

213  size\_t i5,

214  size\_t i6,

215  size\_t i7,

216  const char\* error\_msg,

217  size\_t idx) {

218  check\_range(x.size(),i1,error\_msg,idx);

219  return get\_base1(x[i1 - 1],i2,i3,i4,i5,i6,i7,error\_msg,idx+1);

220  }

221

222

244  template <typename T>

245  inline const T&

246  get\_base1(const std::vector<std::vector<std::vector<std::vector<std::vector<std::vector<std::vector<std::vector<T> > > > > > > >& x,

247  size\_t i1,

248  size\_t i2,

249  size\_t i3,

250  size\_t i4,

251  size\_t i5,

252  size\_t i6,

253  size\_t i7,

254  size\_t i8,

255  const char\* error\_msg,

256  size\_t idx) {

257  check\_range(x.size(),i1,error\_msg,idx);

258  return get\_base1(x[i1 - 1],i2,i3,i4,i5,i6,i7,i8,error\_msg,idx+1);

259  }

260

261

262

282  template <typename T>

283  inline Eigen::Matrix<T,1,Eigen::Dynamic>

284  get\_base1(const Eigen::Matrix<T,Eigen::Dynamic,Eigen::Dynamic>& x,

285  size\_t m,

286  const char\* error\_msg,

287  size\_t idx) {

288  check\_range(x.rows(),m,error\_msg,idx);

289  return x.block(m-1,0,1,x.cols());

290  }

291

308  template <typename T>

309  inline const T&

310  get\_base1(const Eigen::Matrix<T,Eigen::Dynamic,Eigen::Dynamic>& x,

311  size\_t m,

312  size\_t n,

313  const char\* error\_msg,

314  size\_t idx) {

315  check\_range(x.rows(),m,error\_msg,idx);

316  check\_range(x.cols(),n,error\_msg,idx + 1);

317  return x(m - 1, n - 1);

318  }

319

334  template <typename T>

335  inline

336  const T& get\_base1(const Eigen::Matrix<T,Eigen::Dynamic,1>& x,

337  size\_t m,

338  const char\* error\_msg,

339  size\_t idx) {

340  check\_range(x.size(),m,error\_msg,idx);

341  return x(m - 1);

342  }

343

358  template <typename T>

359  inline const T&

360  get\_base1(const Eigen::Matrix<T,1,Eigen::Dynamic>& x,

361  size\_t n,

362  const char\* error\_msg,

363  size\_t idx) {

364  check\_range(x.size(),n,error\_msg,idx);

365  return x(n - 1);

366  }

367

368  }

369 }

370 #endif

stan::math::get\_base1

const T & get\_base1(const std::vector< T > &x, size\_t i, const char \*error\_msg, size\_t idx)

Return a reference to the value of the specified vector at the specified base-one index...

**Definition:** get\_base1.hpp:27

Eigen.hpp

stan::math::check\_range

void check\_range(size\_t max, size\_t i, const char \*msg, size\_t idx)

**Definition:** check\_range.hpp:30

check\_range.hpp


---

[ Stan Home Page ]

*© 2011–2014,
Stan Development Team.*
